# Alternative splicing in stem cells and development: research progress and emerging technologies

**DOI:** 10.1186/s13619-025-00238-w

**Published:** 2025-06-04

**Authors:** Yan Jin, XiaoLin Liang, Xiangting Wang

**Affiliations:** 1 Department of Geriatrics, Gerontology Institute of Anhui Province, The First Affiliated Hospital, Division of Life Sciences and Medicine, University of Science and Technology of China, Hefei, Anhui 230026 China; 2 The RNA Institute, University of Science and Technology of China, Hefei, Anhui 230026 China; 3 Centre for Leading Medicine and Advanced Technologies of IHM, University of Science and Technology of China, Hefei, Anhui 230026 China; 4 MOE Key Laboratory for Membraneless Organelles and Cellular Dynamics, Hefei National Science Center for Physical Sciences at Microscale & University of Science and Technology of China, School of Life Sciences/Division of Biomedical Sciences, Hefei, Anhui 230026 China

**Keywords:** Alternative splicing, Splicing factor, Stem Cells, Differentiation, Development

## Abstract

Alternative splicing is a key regulatory mechanism that generates transcriptomic diversity by selectively splicing pre-RNA molecules in different ways, leading to the production of multiple RNA isoforms from a single gene. This process is crucial for the fine-tuning of gene expression and is tightly regulated during various biological processes. Recent studies have highlighted how alternative splicing contributes to stem cells self-renewal and differentiation, as well as how dysregulation of splicing factors can impact stem cells behavior and lead to developmental abnormalities or diseases. This review summarizes the current understanding of alternative splicing in stem cells and development, focusing on the molecular mechanisms that govern alternative splicing regulation, the role of splicing factors, and the impact of splicing isoforms on stem cell fate determination and developmental processes. We also discuss emerging technologies, such as CRISPR/Cas-based tools, single-cell long-read RNA sequencing, imaging technologies and 3D culture systems, which are advancing our ability to study alternative splicing in vitro and in vivo. Overall, this field is rapidly evolving, revealing new insights into how alternative splicing shapes the molecular landscape and functions of stem cells and developmental processes.

## Background

Alternative splicing (AS) is a fundamental post-transcriptional regulatory mechanism that significantly contributes to the complexity of the transcriptome (Chen & Manley 2009; Fu & Ares 2014; van den Hoogenhof et al. 2016). Over 95% of multi-exon genes in the human genome undergo some form of AS(Pan et al. 2008). This regulatory process allows for the generation of RNA isoforms with distinct functional properties, contributing to cellular adaptability and diversity (Baralle & Giudice 2017).

AS plays a critical role not only in normal cellular functions but also in various biological processes, especially in stem cells and tissue development (Baralle & Giudice 2017; Cui et al. 2024). AS plays a key role in preserving self-renewal and pluripotency of stem cells, and it also ensures their differentiation into specific cell types with spatiotemporal precision during tissue development (Baralle & Giudice 2017; Cui et al. 2024). AS is highly dynamic and involves a series of cis-elements and trans-acting splicing factors that determine the usage of specific splice sites (Ule & Blencowe 2019). Splicing factors such as serine/arginine-rich proteins (SR proteins) and heterogeneous nuclear ribonucleoproteins (hnRNPs) associated with RNA-specific sequence motifs to regulate the AS of key factors, signaling pathway regulators and enzymes, play crucial roles in maintaining stem cells self-renewal and pluripotency, differentiation and tissue development (Baralle & Giudice 2017; Cui et al. 2024; Ule & Blencowe 2019).

Advances in molecular biology and genomics have significantly enhanced our understanding of AS. Single-cell long-read RNA sequencing, now enables the precise mapping of RNA splicing patterns across different cell types, developmental stages, and disease contexts (Joglekar et al. 2024; Kumari et al. 2024). Additionally, clustered regularly interspaced short palindromic repeats (CRISPR)/Cas systems provides diverse tools to manipulate splicing factors and specific splicing events, facilitating the study of how splicing regulation influences stem cell fate determination and development (Du et al. 2020; Hsu et al. 2019; Mehlferber et al. 2023). These technological advancements not only drive our understanding of AS mechanisms but also offer new avenues for disease treatment (Cui et al. 2024; Du et al. 2020; Hsu et al. 2019).

This review will summarize the mechanisms of AS and its roles in stem cells and tissue development. We will focus on how AS regulates self-renewal and differentiation of stem cells and its impact on tissue development. Moreover, we will discuss the applications of emerging technologies like CRISPR/Cas-based tools and single-cell long-read RNA sequencing in AS research. By examining these emerging technologies, we aim to provide a comprehensive view of how AS research is evolving in stem cells and development and its potential therapeutic applications in disease treatment.

## Mechanisms of alternative splicing

RNA splicing occurs in the nucleus, where introns are removed from pre-RNA transcripts, and the exons are joined together to form mature RNA. This process is executed by the spliceosome, a dynamic ribonucleoprotein complex composed of five small nuclear RNAs (snRNAs) and associated proteins, which assemble into small nuclear ribonucleoproteins (snRNPs). The major spliceosome includes U1, U2, U5, and the U4/U6 di-snRNP, while the minor spliceosome comprises U11, U12, U5, and U6atac/U4atac snRNPs. The exon–intron boundaries are defined by the recognition of the 5′ splice site by U1 snRNP, the binding of splicing factor 1 to the branch point site, and the binding of U2 auxiliary factors to the polypyrimidine tract and the 3′ splice site, respectively. This promotes binding of the U2 snRNP at the 3′ intronic region with subsequent assembly of the U4/U6/U5 tri-snRNP for the activation of a catalytic complex, thus performing the two transesterification reactions ligating the two exon sequences and removing the intron in the lariat form (Beusch et al. 2024; Martínez-Lumbreras et al. 2024; Wan et al. 2020).

AS refers to the capacity of a single pre-RNA molecule to undergo multiple splicing events, thereby generating distinct RNA isoforms. AS types include exon skipping, alternative 5′/3′ splice site selection, intron retention and mutually exclusive exons. This process is influenced by cis-elements and trans-acting splicing factors, which together ensure accurate and dynamic control of gene expression. Cis-elements are specific sequences within the pre-RNA that serve as binding platforms for splicing factors, including exonic splicing enhancers and intronic splicing enhancers, which promote spliceosome assembly and induce exon exclusion, as well as exonic splicing silencers and intronic splicing silencers, which repress splicing and induce exon skipping or intron retention. Additionally, conserved sequences such as the 5′ splice site, 3′ splice site, branch point site, and polypyrimidine tract are critical for defining exon–intron boundaries and recruiting the spliceosome. Trans-acting splicing factors are proteins or RNA molecules that bind to these cis-elements to modulate splicing, including activators such as SR proteins, which enhance splicing by recruiting core spliceosome components, and repressors such as hnRNPs, which inhibit splicing by blocking spliceosome access. The general rules of AS regulation involve the combinatorial action of multiple cis-elements and splicing factors, where the balance between activators and repressors determines the splicing outcome. Furthermore, the spatial arrangement of cis-elements, the concentration and post-translational modifications of splicing factors, and the cellular context collectively fine-tune splicing decisions, ensuring proper gene expression and cellular function (Beusch et al. 2024; Martínez-Lumbreras et al. 2024; Wan et al. 2020).

## Alternative splicing in stem cells

Stem cells possess the unique ability to self-renew, maintaining their undifferentiated state through cell division (Cheong & Lufkin 2011; Varzideh et al. 2023). They can also differentiate into a variety of specialized cell types that contribute to development, a property that, in embryonic stem cells (ESCs), is named pluripotency (Baralle & Giudice 2017; Cui et al. 2024). The balance between self-renewal and differentiation is crucial for stem cells fate determination and development (Baralle & Giudice 2017; Cheong & Lufkin 2011; Cui et al. 2024; Varzideh et al. 2023). Recent studies have shown that comprehensive splicing repression, induced by the splicing inhibitor pladienolide B, drives a pluripotent-to-totipotent state transition in mouse ESCs and human ESCs/induced pluripotent stem cells (Li et al. 2024a; Shen et al. 2021). The regulation of AS is essential for maintaining the balance between self-renewal and differentiation in stem cells (Cui et al. 2024; Mehlferber et al. 2023). Specifically, distinct AS events have been shown to modulate key factors, signaling pathway regulators, and enzymes that are integral to the regulatory networks governing stem cell self-renewal and differentiation (Cui et al. 2024; Mehlferber et al. 2023)(Table 1).

**Table 1:** The key factors, signaling pathway regulators, and enzymes as alternative splicing targets in stem cells

Gene	Regulator	AS Type	Splicing Isoforms	Function	Molecular Mechanism	Ref
DPF2	PTBP1	Exon skipping	DPF2-SDPF2-L	Maintenance of ESCs and neuronal differentiation	BAF complexes-chromatin organization-transcription axis	Nazim et al. 2024
Puf60	Rbm38	Exon inclusion	Puf60+Exon5Puf60−Exon5	Neuronal differentiation from ESCs and NPCs to mature neurons	Rbm38/Ptbp1-Puf60−Exon5/Fubp/HnRNP axis	Han et al. 2022
TCF3	hnRNP H/F/A1 PTBP1ESRG	Mutually exclusive exons	E12 (Short form)E47(Long form)	Lineage formation of ESCs	Wnt pathway	Xie et al. 2024 Yamazaki et al. 2019 Yamazaki et al. 2018
Gm2694	/	Exon inclusion	Trincr1PM, Synage	Self-renewal of ESCs, cerebellum development, cell identity, and synaptic integrity	FGF/ERK pathway, Cbln1 expression, and assembly of the LRP1-HSP90AA1-PSD95 complex	Li et al. 2019Jin et al. 2021Wang et al. 2021
NUMB	SRSF2	Exon skipping	NUMB+Exon9 (Long form)NUMB−Exon9 (Short form)	Differentiation of human mesodermal cells	SRSF2-NUMB-NOTCH pathway	Li et al. 2021a, b
Numb	Pqbp1	Exon inclusion	NUMB+Exon9 (Long form)NUMB−Exon9 (Short form)	Proliferation of striatal progenitors	Pqbp1-Numb axis	Liu et al. 2023
Numb	Rbfox3	Exon inclusion	Numb+Exon12Numb−Exon12	Neuronal differentiation of P19 cells and the developing spinal cord	Rbfox3-Numb axis	Kim et al. 2013
Pcmt	Barc	Intron retention	Full-length BarcBarc+Intron1	NPCs lineage progression	Barc/U2 snRNP complex-Pcmt axis	Abramczuk et al. 2017
SULT4A1	MBNL-1/2 CELF-1/2	Exon skipping	Unstable truncated SULT4A1 (+pseudo-exon 6p)Full-length SULT4A1	Differentiation of SH-SY5Y cells, human inducedpluripotent stem cells and mouse embryonic tissue	Lead to stable SULT4A1 protein expression	Idris et al. 2019

### Key factors as AS targets

DPF2 is a subunit of BRG1/BRM-associated factor chromatin remodeling complexes. Exon 7 of *DPF2* is spliced in a tissue-specific and developmental-stage-specific manner. Highly expressed splicing factor PTBP1 controls skipping of exon 7 in *DPF2* to produce the *DPF2-S* isoform required for self-renewal and pluripotency maintenance of ESCs. However, in neuronal progenitor cells (NPCs) or mature neurons, the low or absent PTBP1 resulted in the predominant expression of the *DPF2-L* isoform to induce cell differentiation. The switch in *DPF2* splicing retargets BAF complexes from enhancers to promoters, impacting chromatin organization during ESCs to neuronal differentiation (Nazim M et al. 2024, Li et al. 2021a, b).

Puf60 is a nucleic acid-binding protein that plays a role in AS and transcription regulation. Recently, a systematic exploration of dynamic neurogenesis splicing networks revealed splicing factor Rbm38 interacts with Ptbp1 to negatively regulate neuronal AS, thereby maintaining the status of ESCs. Down-regulation of Rbm38 promotes inclusion of exon 5 in *Puf60*, which induces neuronal differentiation from ESCs, NPCs, to mature neurons (Han et al. 2022).

Moreover, many AS events of key factors orchestrated by splicing factors are essential for stem cell fate determination, including: HNRNPH1/H2-*TRF2 *(Grammatikakis et al. 2016), SRRM4-*Zfyve27 *(Ohnishi et al. 2017), Ptbp1-*Pbx1 *(Linares et al. 2015), Prpf31-DNA repair and spindle assembly associated factors (Li et al. 2021a), YBX1-osteogenic differentiation and senescence related factors (Xiao et al. 2023), DDX21-key pro-differentiation factors (Miao et al. 2023), SOX9-*SRSF5 *(Puri et al. 2024), SF3B1-mitotic factors axis (Sarchi et al. 2024), among others.

### Signaling pathway regulators as AS targets

A genome-wide analysis of differentiating mouse ESCs has revealed that AS is predominantly associated with Wnt signaling pathways. Selective knockdown of distinct isoforms of the Wnt transcription factor Tcf3 demonstrated they have unique targets for transcriptional repression, leading to diverse cell fates in ESCs (Salomonis et al. 2010). A number of subsequent studies have found splicing factor hnRNP H/F/A1, PTBP1 and long noncoding RNA ESRG co-regulate the selection of mutually exclusive exon of TCF3, thereby determining the self-renewal or differentiation of ESCs (Xie et al. 2024; Yamazaki et al. 2019, 2018).

The long noncoding RNA *Gm2694* exhibits high expression in the ESCs and cerebellum, and is significantly regulated by AS. The ESCs-enriched splicing isoform *Trincr1* associates with TRIM71 to repress FGF/ERK signaling, thereby promoting the self-renewal of ESCs. Additionally, *Trincr1* could repress FGF/ERK signaling and promote differentiation of NPCs into mature neuron cells (Li et al. 2019).

The endocytic adaptor protein NUMB is a pivotal regulator of the NOTCH signaling pathway, with its AS events playing a decisive role in steering the fate determination of various stem cell types. Li et al*.* found that *NUMB* is predominantly expressed as the *NUMB* + *Exon9* long isoform in human APLNR^+^ mesoderm cells. However, During APLNR^+^ mesoderm differentiation, the splicing factor SRSF2 inhibits inclusion of exon 9 in *NUMB* to produce *NUMB − Exon9* short isoform. This short isoform specifically governs the NOTCH pathway and induces the formation of endothelial progenitor cells from human mesodermal cells (Li et al. 2021b). Similarly, Liu et al*.* highlighted the involvement exon 9 of *Numb* in striatal progenitors proliferation, where splicing factor Pqbp1 associates with splicing machinery components and promotes inclusion of exon 9 in *Numb* to induce striatal progenitors proliferation (Liu et al. 2023). Furthermore, Kim et al*.* demonstrated that Rbfox3/NeuN represses inclusion of exon 12 in *Numb *via interaction with a conserved UGCAUG motif, promoting neuronal differentiation in P19 cells and the developing chicken spinal cord (Kim et al. 2013).

In addition to the three aforementioned signaling pathways, the NF-κB pathway (Choudhary et al. 2022), PI3K-AKT pathway (Du et al., 2023; Rademacher et al. 2025; Tan et al. 2019), MAPK pathway (Du et al. 2023; Wheeler et al. 2022), JAK/STAT pathway (Willekens et al. 2023), and TGFβ pathway (Muench et al. 2018) are also implicated in the role of AS regulation in controlling the fate determination of various stem cells.

### Enzymes as AS targets

The *Pcmt* gene encodes a protein belonging to the type II class of protein carboxyl methyltransferase enzymes. Diseases associated with *PCMT* include Alzheimer's disease and spina bifida. The evolutionarily conserved splicing co-factor Barricade (Barc)/Tat-SF1/CUS plays a crucial role in NPCs lineage formation in *Drosophila* brain development (Abramczuk et al. 2017). Mechanically, Barc associates with components of the U2 snRNP complex to orchestrate intron retention in *Pcmt* and other neuronal genes in specific genes (Abramczuk et al. 2017).

*SULT4A1* is a highly conserved sulfotransferase-like gene across species. Studies on *Sult4a1* knockout mice have revealed that this protein is crucial for normal development, with its absence leading to a severe neurological phenotype. In human tissues, there are two transcripts of *SULT4A1*: one produces a full-length protein, while the other yields an unstable truncated protein due to the inclusion of a pseudo-exon (6p) between exons 6 and 7. The transcript containing the pseudo-exon 6p is widely expressed, whereas the transcript producing the full-length protein has a more restricted expression pattern. Splicing factors such as MBNL-1/2 and CELF-1/2 play a significant role in promoting exclusion of pseudo-exon 6p in *SULT4A1* during neuronal differentiation. This process involves the removal of the pseudo-exon, resulting in stable protein expression. This regulatory mechanism has been demonstrated in SH-SY5Y cells, human induced pluripotent stem cells, and mouse embryonic tissue (Idris et al. 2019).

Beyond the enzymes previously discussed, emerging evidence highlights the functional significance of splicing factor-enzyme partnerships in governing stem cell fate determination through AS regulation, including FUBP1-*KDM1A/LSD1 *(Hwang et al. 2018), Dhx38-cell cycle kinases (Tu et al. 2022), Dppa5a-*Rev1*and *Polq *(Jiang et al. 2023), and GPATCH8-*MAP3K7 *(Benbarche et al. 2024).

### The mechanisms driving functional differences of AS isoforms

Although numerous studies have explored the functional differences of AS isoforms, the molecular mechanisms driving these differences remain largely elusive. Different isoforms exhibit functional diversity possibly due to variations in structure, localization, stability, and interaction networks. These variations can result from the inclusion or exclusion of key functional domains, changes in subcellular localization signals, exposure or masking of binding sites, alterations in stability mediated by degradation signals, and modifications in non-coding regions that influence RNA stability and translation efficiency. For example, the aforementioned NUMB protein consists of amino-terminal phospho-tyrosine-binding domain, proline-rich domain, and Eps 15 homology region. The phospho-tyrosine-binding domain typically facilitates interactions with the NPxY motif in transmembrane proteins. proline-rich domain, found in certain isoforms, contain Src homology binding sites crucial for intracellular signal transduction. NUMB also interacts with intracellular adapters like alpha-adaptin and Eps15 via its Eps 15 homology region. Aforementioned NUMB isoforms differing in exon 9 or 12 inclusion within the proline-rich domain exhibit distinct functions, likely due to structural domain alterations (Choi et al. 2021).

### The regulatory mechanisms of splicing factors

Various studies have demonstrated dynamic alterations in the expression and subcellular localization of splicing factors (e.g., MBNL proteins, SR proteins, LSM proteins, DDX proteins, and SNRP proteins) during stem cell differentiation, embryonic development, tissue morphogenesis, and signaling pathway transitions, where these splicing factors play critical roles in regulating gene expression and cellular functions (Han et al. 2013; Li et al. 2025; Sanford & Bruzik 2001; Solana et al. 2016). these splicing factors are frequently dysregulated across multiple tumor types, where they drive oncogenic processes such as uncontrolled proliferation, aberrant differentiation, reduced apoptosis, increased migration, enhanced metastatic potential, chemotherapy resistance, and immune evasion (Bradley & Anczuków, 2023; Dho et al. 2025; Hernández et al. 2016). Furthermore, the upstream regulation of splicing factors involves multiple mechanisms: (1) a mutual regulatory network between splicing factors and transcription factors(Even-Ros et al. 2025); (2) reciprocal regulation of AS events among splicing factors (Jangi et al. 2014); (3) autoregulatory negative feedback loops, where splicing factors bind their own pre-mRNAs to induce nonsense-mediated decay (Jangi et al. 2014); (4) upstream control by non-coding RNAs, which fine-tune splicing factor expression or activity (Liu et al. 2021; Mehlferber et al. 2023); and (5) Coupling of transcriptional and chromatin states (e.g. histone modifications and DNA methylation) with AS (Yustis et al. 2024). Collectively, these layered regulatory circuits highlight the centrality of splicing plasticity in stem cells, development and associated diseases.

## Alternative splicing in development

During development, AS are tightly controlled to ensure proper gene expression patterns that dictate cell fate determination, tissue formation, and organogenesis (Baralle & Giudice 2017; Olthof et al. 2022). Multiple transcriptome sequencing analyses have revealed that AS events and splicing factors are differentially expressed at key stages in the development of various tissues, and some of the splicing factors exhibit tissue or cell types-specific expression patterns (Li et al. 2024b; Saito et al. 2019). To date, numerous splicing factors and specific AS events have been shown to play different functions in various tissue developmental processes. Among various tissues, the brain and heart exhibit the most tissue-specific AS expression patterns and have been the most extensively researched in terms of the developmental and physiological effects of AS (Buljan et al. 2012; Ellis et al. 2012; Weskamp et al. 2021).

### AS in brain development

**Fig. 1:**
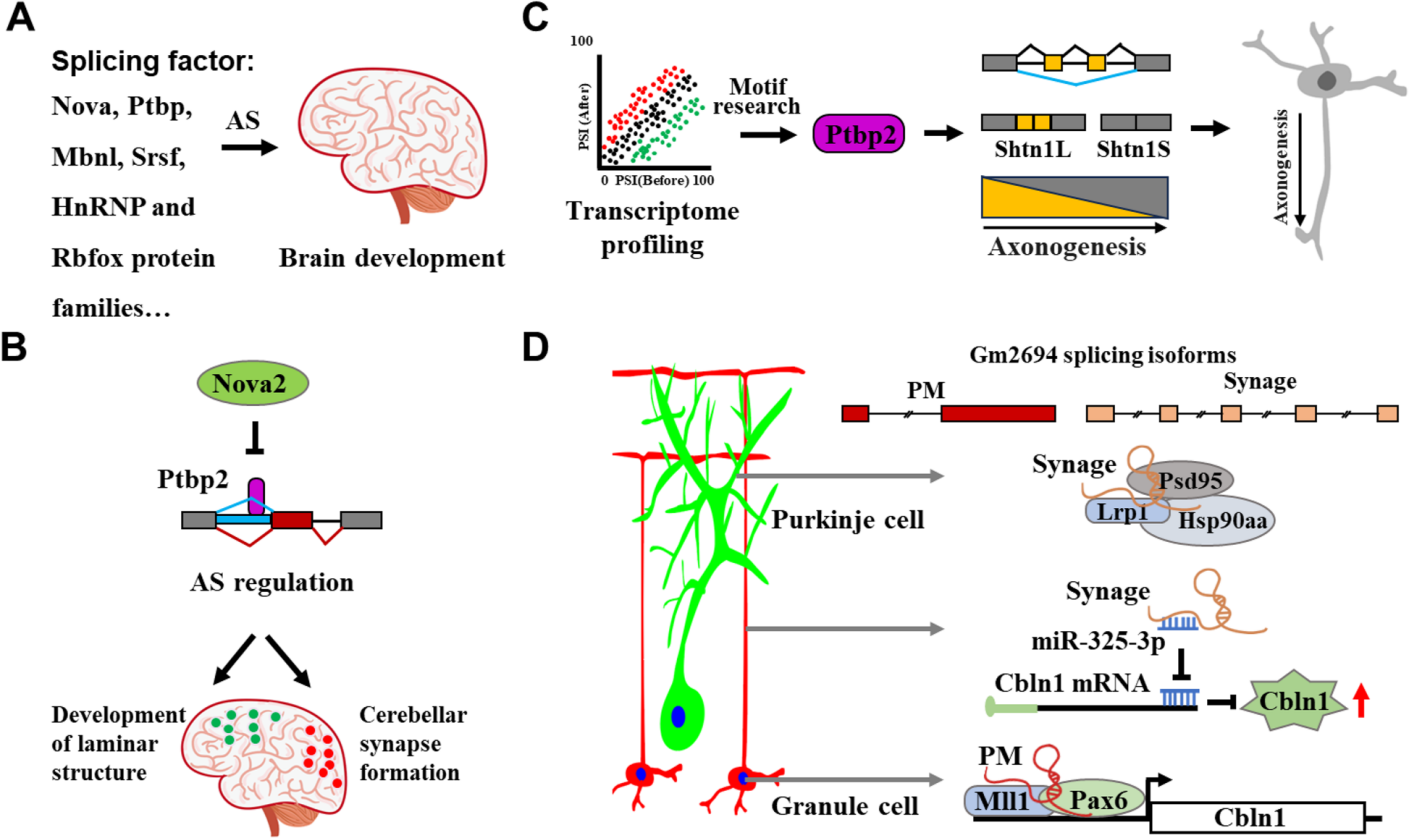
Splicing factors and specific AS events in brain development. **A** Neuron-enriched splicing factor protein families co-regulate brain development. **B** Nova2 represses exon inclusion and Ptbp2-mediated intron retention to control specifically development of laminar structure in cortical excitatory neurons (Green dots) as well as proper motor coordination and synapse formation in cerebellar inhibitory Purkinje cells (Red dots). **C** Ptbp2, identified by transcriptome profiling, promotes exon skipping in *Shtn1* during axonogenesis. **D** The long noncoding RNA *Gm2694* undergoes AS to generate multiple isoforms in the cerebellum, including *PM* and *Synage*. *PM* is enriched in cerebellar granule cells. It associates with the Pax6/Mll1 complex to enhance transcription of *Cbln1* and synapse integrity in the nucleus of granule cells. *Synage* is predominantly expressed in cerebellar Purkinje cells. It regulates synapse stability and neuronal function by acting both as a scaffold for the Lrp1-Hsp90aa-Psd95 complex in postsynaptic terminals of Purkinje cells and as a miRNA sponge in presynaptic terminals of granule cells

Studies have showed neuron-enriched splicing factors, including Nova, Ptbp, Mbnl, Srsf, HnRNP and Rbfox protein families, co-regulate the development of distinct brain regions and neuronal subtypes (Baralle & Giudice 2017) (Fig. 1A). These splicing factors not only regulate the proliferation and differentiation of NPCs as described above, but also cell identity, axon growth and guidance, synapse formation, synapse specificity, and many other functions. Nova2 acts as an essential splicing factor that specifically regulates laminar structure development in cortical excitatory neurons as well as proper motor coordination and synapse formation in cerebellar inhibitory Purkinje cells. Nova2 leads to different outcomes in distinct neuronal subtypes on the same transcripts and prevents introns retention serving as cis-acting scaffolds for Ptbp2. *Nova* knockout mice have aberrant AS patterns of many neuromodulator genes such as *Gphn*, *Itpr1*, *Slc8a1*, and *Arhgap2 *(Saito et al. 2019) (Fig. 1B). Through unbiased transcriptome profiling of immature primary cortical neurons during early axon formation, Zhang et al*.* discovered an association between axon guidance and neuron-specific AS events. Ptbp2 exhibits neuronal enrichment with expression peaking during the axon formation phase in developing brains. Cortical depletion of *Ptbp2* specifically disrupts AS patterns of axon associated genes and axon formation. Ptbp2 modulates AS of axon-associated gene *Shtn1*, thereby modulating actin interaction, polymerization, and axon growth (Zhang et al. 2019) (Fig. 1C).

Recently, AS of long noncoding RNAs have been found to be associated with cell identity and brain development. The conserved highly expressed long noncoding RNA *Gm2694* has more than 10 splicing isoforms in the cerebellum. *Gm2694* is located near the *Cbln1* gene, a synaptic organizer protein that specifically directs axon growth, guidance and synapse formation between cerebellar granule cells and Purkinje cells. One of these isoforms of *Gm2694*, *PM*, is enriched in cerebellar granule cells. We have revealed that *PM* enhances transcription of *Cbln1* in an isoform-specific manner through the Pax6-Mll1-H3K4me3 axis and maintains cerebellar synaptic integrity (Jin et al. 2021). In contrast, another reported *Gm2694* AS isoform, *Synage*, is predominantly expressed in cerebellar Purkinje cells. It regulates synapse stability and neuronal function by acting both as a scaffold for Lrp1-Hsp90aa-Psd95 complex and as a miRNA sponge (Wang et al. 2021) (Fig. 1D).

### AS in heart development

**Fig. 2:**
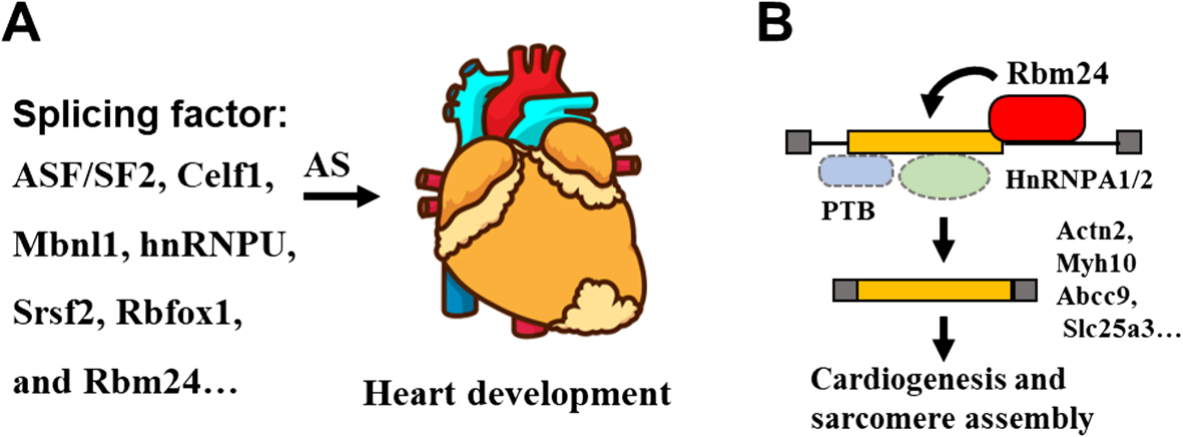
Splicing factors and specific AS events in heart development. **A** Various splicing factors mediate specific AS events to co-regulate heart development. **B** Rbm24 prevents PTB- and hnRNP A1/A2-mediated suppression of exon inclusion. Rbm24 facilitates exon inclusion in genes such as *Actn2*, *Myh10*, *Abcc9*, and *Slc25a3* to control cardiogenesis and sarcomere assembly

Just as occurs in the brain, splicing factors also participate in both collaborative and opposing actions during the development of heart (van den Hoogenhof et al. 2016). In the process of heart development, there is a significant alteration in the levels of expression of various splicing factors such as ASF/SF2 (Xu et al. 2005), Celf1 (Kalsotra et al. 2008), Mbnl1 (Kalsotra et al. 2008), Srsf2 (Zhang et al. 2015), hnRNPU (Ye et al. 2015), Rbfox1 (Gao et al. 2016), and Rbm24 (de Groot et al. 2020; Lu et al. 2022; Saquet et al. 2024). These splicing factors, by mediating specific AS events, have been linked to vesicular trafficking, membrane and cytoskeleton remodeling, ion channel function, and chromatin modifications in cardiac ventricles, cardiomyocytes, and fibroblasts (Giudice et al. 2014; van den Hoogenhof et al. 2016; Wang et al. 2015) (Fig. 2A). Deletion of these genes in mice leads to significant cardiac developmental malformation (van den Hoogenhof et al. 2016). For example, mice lacking *Rbm24* die between embryonic day 12.5 and embryonic day 14.5 because of multiple cardiac malformations, including ventricular septum defects, reduced trabeculation and compaction, and dilated atria (de Groot et al. 2020; Lu et al. 2022; Saquet et al. 2024). Mechanistically, transcriptomics revealed the cardiogenesis and sarcomere assembly related genes including *Actn2*, *Myh10*, *Abcc9*, and *Slc25a3* were mis-spliced in mouse heart lacking *Rbm24*. Rbm24 prevents PTB- and hnRNP A1/A2-mediated suppression of exon inclusion. Rbm24 facilitates inclusion of exon 6 in *Actn2* at distinct stages of cardiac differentiation and is crucial to sarcomere assembly and integrity (Lu et al. 2022; Saquet et al. 2024) (Fig. 2B).

In addition to brain and heart development, AS plays an essential role in other tissues or organs such as skeletal muscle, liver, testis, and the hematopoietic system. These reports conclude that AS is an indispensable regulator of tissue development (Baralle & Giudice 2017; Olthof et al. 2022).

## Emerging technologies in alternative splicing research

**Fig. 3:**
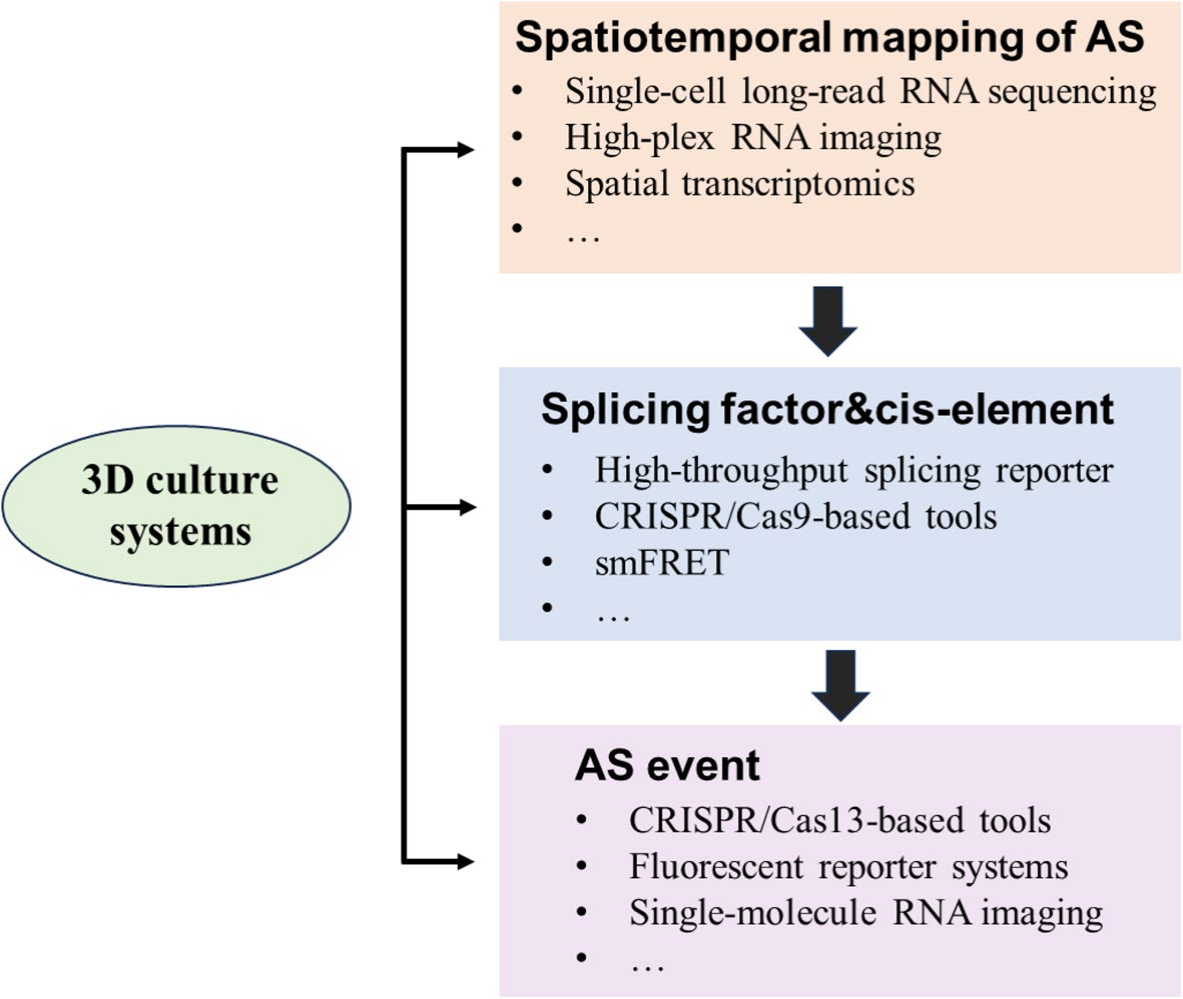
Emerging technologies in AS research

Emerging technologies in AS research have profound implications for understanding stem cells and development. In addition to traditional techniques such as RNA immunoprecipitation and sequencing (RIP-seq), crosslinking-immunoprecipitation and sequencing (CLIP-seq), and splicing factor interactome by mass spectrometry, several cutting-edge technologies are advancing our understanding of AS in stem cells and development (Fig. 3).

### Editing/manipulation technologies

The CRISPR/Cas system, widely known for its genome-editing capabilities, can also be adapted to modify RNA splicing through precise interventions (Hsu et al. 2019). CRISPR/Cas9 system can be used to target splicing factor genes or cis-elements and dissect their role in stem cells, development and associated diseases (Hsu et al. 2019). CRISPR interference and CRISPR activation allow for the fine-tuned modulation of splicing factor expression without altering DNA sequences (Du et al. 2020). Moreover, the development of CRISPR/Cas13, a tool for targeting RNA directly, allows researchers to manipulate specific AS event without modifying the underlying DNA sequence, offering a reversible and dynamic approach to studying AS regulation in living cells (Kordys et al. 2022). For example, Du et al*.* have developed CRISPR artificial splicing factors (CASFx), which consist of catalytically inactive dCasRx or dPspCas13b fused to splicing regulatory domains from RBFOX1 and RBM38. Their study demonstrated that by designing guide RNA to target specific sites within transcripts, CASFx could precisely modulate AS event, achieving two-fold to 20-fold changes in exon inclusion or exclusion compared to controls. This system enables programmable and reversible "rewriting" of AS events. Furthermore, the researchers developed rapamycin-inducible CASFx and implemented multiplexed targeting using orthogonal dCas13 variants. As proof of therapeutic potential, they successfully restored the inclusion of exon 7 in *SMN2* in patient-derived spinal muscular atrophy fibroblasts (Du et al. 2020). Presently, the traditional CRISPR/Cas9 system has been extensively utilized in large-scale splicing factor screens to identify master regulators governing specific AS events in stem cells, development and associated diseases (Kordys et al. 2022). The CRISPR interference, CRISPR activation and CRISPR/Cas13 systems are also gradually being utilized in some AS regulation studies, but the effects need to be verified by more studies (Kordys et al. 2022).

### Sequencing technologies

Long-read RNA sequencing technology, such as Nanopore and Pacbio, has proven invaluable in studying RNA splicing (Kumari et al. 2024). Unlike traditional short-read RNA-seq, long-read RNA sequencing directly detects long-read transcripts, including complex splicing events, splicing isoforms, and novel RNA species (Kumari et al. 2024). This capability significantly improves the reliability of AS analysis because it provides a comprehensive view of transcript structures without the need for assembly from shorter reads, thereby reducing ambiguities and artifacts associated with traditional short-read approaches. Moreover, single-cell long-read RNA sequencing is a powerful approach that allows for the detailed study of gene expression at both the individual cell and RNA isoform level. This combination provides a comprehensive view of the transcriptome, including AS pattern, isoform diversity, and gene regulation in stem cells, development and associated diseases (Joglekar et al. 2024; Kumari et al. 2024). For example, Joglekar et al*.* have performed single cell isoform RNA sequencing 2 to investigate brain-region-specific, cell-type-specific and developmental-stage-specific isoform regulation. They found full-length isoform expression varies along one or more axes in 72% of genes. AS differs significantly across cell types, influencing protein architecture and disease-linked variation. Neurotransmitter transport and synapse-related genes also exhibit cell-type variability across anatomical regions. AS is particularly dynamic during postnatal day 21–28 in mice, with developmental regulation outweighing regional differences (Joglekar et al. 2024). Despite their advantages, long-read RNA sequencing has limitations including higher error rates, lower throughput, and immature or computationally intensive analysis tools. These drawbacks are particularly pronounced in single-cell applications.

High-throughput splicing reporter assays have become essential tools for large-scale screening of cis-elements and trans-acting factors. These assays typically involve engineered constructs containing AS exons coupled to fluorescent or luminescent markers, allowing for real-time measurement of splicing outcomes (Rhine et al. 2019). These reporter systems are used to study how splicing changes in stem cells, development and associated diseases, and to discover novel cis-elements or trans-acting factors involved in these changes (Rhine et al. 2019). For example, Benbarche et al*.* have employed a synthetic intron-based reporter system in a genome-wide CRISPR screen to identify suppressors of *SF3B1* mutation-induced mis-splicing. This approach revealed GPATCH8 as a critical effector of mutant *SF3B1*-dependent splicing dysregulation. Strikingly, *GPATCH8* knockdown rescued about 30% of aberrant splicing events caused by *SF3B1* mutations and ameliorated defective hematopoiesis in both *SF3B1*-mutant murine models and primary human hematopoietic progenitors, suggesting its therapeutic potential (Benbarche et al. 2024). However, this approach has primarily been applied to cellular systems and large-scale splicing factor screening, while its utility for in vivo studies and genome-wide identification of cis-elements remains limited, necessitating further methodological advancements.

### Imaging technologies

Imaging technologies have become indispensable for dissecting the spatiotemporal dynamics of AS in stem cells and developmental research. Single-molecule fluorescence resonance energy transfer (smFRET) combined with super-resolution microscopy techniques including stimulated emission depletion (STED) and photoactivated localization microscopy (PALM) reveals the nanoscale spatial organization of splicing factors within the nucleus and their interactions with the spliceosome (Ha et al. 2024). Fluorescent reporter systems (e.g., dual-color GFP/RFP) and single-molecule RNA imaging (e.g., MS2/MCP, Cas9/13-based RNA imaging and fluorescent RNA aptamers) combined with super-resolution microscopy techniques enable real-time monitoring of exon inclusion/skipping ratios or the expression of specific RNA isoforms, allowing the visualization of splicing events in living cells and tissues (Eichenberger et al. 2023). Additionally, high-plex RNA imaging—including multiplexed error-robust fluorescence in situ hybridization (MERFISH) and spatially resolved transcript amplicon readout mapping (STARmap), and spatial transcriptomics (e.g., 10X Genomics Visium and Slide-seq)—enable the capture of RNA isoforms spatial distribution and reveal local intercellular communication networks operating in situ in stem cells and development (Longo et al. 2021). Together, these multimodal imaging approaches are transforming static splicing maps into dynamic, multiscale regulatory networks, providing powerful visualization tools for targeting splicing defects in stem cells and developmental disorders. However, no imaging method currently provides as complete a scope of the AS as does single-cell long-read RNA sequencing, underscoring the need for approaches to integrate single-cell and spatial data.

### Novel models

3D culture systems is a cell culture technology that mimics the microenvironment of tissues or organs in vivo. Unlike traditional 2D monolayer cultures that oversimplify cellular environments and often fail to recapitulate in vivo complexities, 3D cultures allow cells to grow, interact, and form structures that more closely resemble physiological states, such as organoids, spheroids, or tissue-like constructs, in three dimensions. These structures closely mimic the architecture, functionality, and cellular heterogeneity of their native tissues. Unlike traditional model organisms such as mice, organoids derived from human stem cells (e.g., brain, heart, lung, liver, etc.) can reveal the human tissue development, evolution and pathological processes, which is of great significance, especially in the fields of drug screening and regenerative medicine (Bowles et al. 2021; Trujillo et al. 2021; Yang et al. 2023). Presently, 3D culture systems, particularly organoids, have become powerful models for AS studies in stem cells, development and associated diseases (Yang et al. 2023). For instance, brain organoids, meticulously crafted from human pluripotent stem cells, have been instrumental in unraveling the mysteries of neurodevelopment and neurological disorders. Researchers have utilized these cerebral models to demonstrate how precise AS regulation orchestrates the transition from neural progenitors to mature neurons, highlighting the critical role of AS in neurogenesis and brain circuitry formation (Trujillo et al. 2021; Sebastian et al. 2023). Moreover, by introducing mutations associated with Parkinson's disease or other diseases into human pluripotent stem cells prior to organoid differentiation, researchers could observe aberrant AS patterns contributing to pathological processes such as dopaminergic neuron loss, and further enable the exploration of therapeutic interventions, including small-molecule compounds or gene-targeted therapies (Boussaad et al. 2020; Bowles et al. 2021; Chen et al. 2024).

## Conclusions and perspectives

In summary, we have now identified critical roles for AS in stem cell fate determination and development. Despite significant progress, our understanding of the global AS dynamics governing these processes remains fragmented, with the functional mechanisms of most core splicing factors and AS events still elusive, while AS-targeted therapeutic development is only beginning to emerge beyond a few pioneering applications. Therefore, future investigations should focus on at least three key directions: (1) spatiotemporal mapping of AS dynamics through integrated single-cell/spatial multi-omics and epigenetic crosstalk analysis; (2) continued functional and mechanistic dissection of master splicing factor-specific AS event partnerships in stem cell fate determination, development and associated diseases; and (3) development of precision intervention drugs (e.g., small-molecule compounds, CASFx and antisense oligonucleotides) for correcting disease-associated splicing defects in cross-species models, patient-derived organoids and clinical patients.

Nevertheless, critical challenges persist across multiple dimensions: technologically, limitations in long-read sequencing throughput and sensitivity hinder detection of low-abundance isoforms; mechanistically, functional redundancy among splicing factors and microenvironmental influences complicate regulatory network analysis; experimentally, discrepancies between in vitro models and in vivo systems persist, alongside interspecies translatability gaps; and therapeutically, delivery inefficiencies and off-target effects impede clinical translation. Overcoming these hurdles will require multidisciplinary innovations—from advanced sequencing platforms and bioinformatics pipelines to engineered delivery systems and physiologically relevant culture platforms. Successfully addressing these challenges will unlock AS's full potential for deciphering stem cells and developmental programs and advancing medicine applications.

## Data Availability

Not applicable.

## Abbreviations

AS: Alternative splicing
CLIP-seq: Crosslinking-immunoprecipitation and sequencing
CRISPR: Clustered regularly interspaced short palindromic repeats
ESCs: Embryonic stem cells
hnRNP: Heterogeneous nuclear ribonucleoprotein
MERFISH: Multiplexed error-robust fluorescence in situ hybridization
NPCs: Neuronal progenitor cells
PALM: Photoactivated localization microscopy
RIP-seq: RNA Immunoprecipitation and Sequencing
smFRET: Single-molecule fluorescence resonance energy transfer
snRNA: Small nuclear RNA
snRNPs: Small nuclear ribonucleoproteins
SR protein: Serine/arginine-rich protein
STARmap: Spatially resolved transcript amplicon readout mapping
STED: Stimulated emission depletion
